# Temporal Profile of Cerebrovascular Reactivity Impairment, Gray Matter Volumes, and Persistent Symptoms after Mild Traumatic Head Injury

**DOI:** 10.3389/fneur.2016.00070

**Published:** 2016-05-11

**Authors:** Leodante da Costa, Christiaan Bas van Niftrik, David Crane, Jorn Fierstra, Allison Bethune

**Affiliations:** ^1^Division of Neurosurgery, Department of Surgery, Sunnybrook Health Sciences Centre, University of Toronto, Toronto, ON, Canada; ^2^Department of Medical Imaging, Sunnybrook Health Sciences Centre, University of Toronto, Toronto, ON, Canada; ^3^Division of Neurosurgery, University Hospital Zurich, Zurich, Switzerland; ^4^Brain Sciences Program, Sunnybrook Research Institute, Toronto, ON, Canada

**Keywords:** cerebrovascular reactivity, concussion, head injury, traumatic brain injury

## Abstract

**Objective:**

Increased awareness around neurocognitive deficits after mild traumatic brain injury (mTBI) has progressed the search for objective, diagnostic, and monitoring tools, yet imaging biomarkers for mTBI and recovery are not established in clinical use. It has been suggested that mTBI impairs cerebrovascular reactivity (CVR) to CO_2_, which could be related to post-concussive syndrome (PCS). We investigate CVR evolution after mTBI using blood-oxygen-level dependent (BOLD) magnetic resonance imaging (MRI) and possible correlation with PCS.

**Methods:**

A prospective cohort of 25 mTBI patients and 18 matched controls underwent BOLD MRI CVR measurements. A subset of 19 mTBI patients underwent follow-up testing. Visits took place at a mean of 63 and 180 days after injury. Symptoms were assessed with the Sport Concussion Assessment Tool 2 (SCAT2). Symptoms, CVR and brain volume [gray matter (GM), white matter (WM), and whole brain (WB)], age, and sex, were examined between groups and longitudinally within traumatic brain injury (TBI) patients.

**Results:**

Traumatic brain injury participants were 72% males, mean age being 42.7 years. Control participants were 61% with mean age of 38.7 years. SCAT2 scores tended to improve among those mTBI patients with follow-up visits (*p* = 0.07); however, they did not tend to recover to scores of the healthy controls. Brain volumes were not statistically different between groups at the first visit (WM *p* = 0.71; GM *p* = 0.36). In mTBI patients, there was a reduction in GM volume between visits 1 and 2 (*p* = 0.0046). Although mean CVR indexes were similar (WM *p* = 0.27; GM *p* = 0.36; and WB *p* = 0.35), the correlation between SCAT2 and CVR was negative in controls (WM-*r* = −0.59; *p* = 0.010; GM-*r* = −0.56; *p* = 0.016; brain-*r* = −0.58; *p* = 0.012) and weaker and positive in mTBI (brain-*r* = 0.4; *p* = 0.046; GM-*r* = 0.4; *p* = 0.048). SCAT2 correlated with GM volume (*r* = 0.5215, *p* = 0.0075) in mTBI but not in controls (*r* = 0.2945, *p* = 0.2355).

**Conclusion:**

There is a correlation between lower GM CVR indexes and lower performance on SCAT2 in patients with mTBI, which seems to be associated with more symptoms. This correlation seems to persist well beyond 120 days. mTBI may lead to a decrease in GM volume in these patients.

## Introduction

Traumatic brain injury (TBI) is a silent worldwide epidemic. It is estimated that mild injuries represent up to 75–90% of all brain injuries (1–3). Although the vast majority of patients recover well within weeks, up to 30% will suffer with persistent neurocognitive, affective, and psychological symptoms (4), in a condition known as post-concussive syndrome (PCS). Persistent symptoms represent not only a personal burden to individual patients but also a socioeconomic issue, considering that most patients with TBI are relatively young (2).

Despite often disabling symptoms, routine imaging [computerized tomography (CT) and magnetic resonance imaging (MRI)] is frequently normal or non-specific (5). Functional imaging appears to increase the sensitivity for detecting abnormalities (6), and it has been suggested that it might even help to predict outcome in mild traumatic brain injury (mTBI) patients (7). Advances in neuroimaging, especially in MRI techniques, now allow the identification of a subgroup of mTBI patients where microscopic axonal damage occurs despite normal standard imaging. Diffusion tensor imaging (DTI), measuring microstructural changes, suggests that there are indeed significant differences between mTBI patients and healthy individuals even if gross anatomical abnormalities cannot be seen on CT or MRI (8, 9).

The pathophysiological processes behind persistent post-concussive symptoms are not clear. Altered cerebral blood flow (CBF) and disturbance of CBF regulatory mechanisms after mTBI may be related to post-injury symptoms (10). One important mechanism for CBF regulation is cerebrovascular reactivity (CVR), which describes the degree of changes in intracranial vessel diameter in response to chemical stimuli. CVR can be defined as the percentage change in CBF or CBF velocity [if transcranial Doppler (TCD) is used as the measurement tool] over the absolute change in the stimuli. It has been suggested that altered CBF control mechanisms (CVR or autoregulation) following mTBI may help to identify patients at risk of prolonged post-concussive symptoms (11) or secondary injury (12).

Functional MRI (fMRI) based on blood-oxygen-level dependent (BOLD) sequences is able to detect changes in blood flow inferred from changes in concentrations of oxygenated hemoglobin. Therefore, it can be used to investigate CVR if the appropriate stimulus is provided. However, task-based fMRI is based on the assumption that neurovascular coupling is intact, i.e., regional flow will change in response to increased demand. Coupling is affected by many factors and may vary in different regions of the brain, and this assumption might not be correct in head injury (13–16). Recently, Maggio et al. (17) showed that changes in P_a_CO_2_ seem to influence neurovascular coupling but do not affect CVR measured using TCD and 5% carbon dioxide (CO_2_) inhalation. Although not a direct CBF measurement, the BOLD sequence provides a strong surrogate CVR measurement with high spatial resolution and is therefore able to detect small areas with abnormal CVR. Moreover, the use of CO_2_, a potent stimulus of CVR, provides a “global view” of vascular response and has been shown to be the most reproducible stimulus (18).

To investigate the relationship between CVR to CO_2_ and symptoms post-mild brain injury, we used fMRI-BOLD imaging coupled with precise CO_2_ manipulation (19) and a standardized concussion assessment questionnaire [Sport Concussion Assessment Tool 2 (SCAT2)] (20) to evaluate symptom severity in a cohort of mTBI patients at two distinct time points following injury, and a matched control group.

## Materials and Methods

Research Ethics Board approval for the study at Sunnybrook Health Sciences Centre was obtained. A prospective cohort of 25 patients with recently (<3 months) diagnosed mTBI and 18 age- and sex-matched controls were recruited. mTBI was defined according to the criteria of the American Academy of Neurology (21) as a “traumatically induced physiological disruption of brain function,” as manifested by at least one of the following: loss of consciousness ≤30 min; amnesia time ≤24 h; alteration in mental state with a Glasgow Coma Scale (GCS) ≤13 after 30 min from injury; and focal neurological deficit(s) that may or may not be transient.

Patients with history of previous neurological (brain) surgery, previous moderate or severe head injuries or a recent concussion (<1 year), any associated major trauma, mTBI with significant posttraumatic CT changes (contusions, subdural hematomas), severe pulmonary disease, severe carotid stenosis or occlusion, or contraindications for MRI examination were excluded.

### MRI and CO_2_ Challenge

Two MRI scans with identical protocols were obtained at different occasions after injury. Time from injury to first visit was on average 63.6 days and to second visit 180 days. Functional magnetic resonance imaging of the brain (FMRIB) Software Library tools were used on the high resolution T1-weighted images to remove the skull (22) and correct for field inhomogeneities and perform automated segmentation into gray matter (GM), white matter (WM), and cerebrospinal fluid (CSF) (23). Region of interest (ROI) volumes were then extracted from GM and WM regions. MRI BOLD images are acquired with the following parameters: GRE-EPI TR/TE = 2000/30 ms, 255 volumes, flip angle = 90°, 64 × 64 matrix, 3.6 mm × 3.6 mm × 3 mm voxel size, axial (8:38), SPGR 3D sequence for co-registration and segmentation, multi-echo T2 sequence, and 2D FLAIR.

Carbon dioxide was manipulated using prospective targeting of end-tidal CO_2_, as described previously by Slessarev et al. (19) Briefly, a mask connected to the computerized targeting rebreathing system (RespirAct™ – Thornhill Research, Toronto, ON, Canada) is taped over the mouth and nose. A 10-min period allowing acclimatization to wearing the breathing mask while in the MRI scanner is given, while anatomical MRI sequences are acquired. CO_2_ levels are raised 10 mmHg above baseline, kept for 45 s, and then returned to baseline. The sequence is repeated with hypercapnia of 130 and 90 s intervals between the two runs. MRI BOLD sequences are acquired during the time of CO_2_ manipulation.

To obtain the CVR maps, from the tidal pCO_2_ waveforms generated by the RespirAct™, the end-tidal points are manually selected generating end-tidal pCO_2_ waveforms. MR and P_ET_CO_2_ data are imported into the software Analysis of Functional Neuroimaging (AFNI) (24). The first raw images of each BOLD MRI acquisition are reviewed, and the first three volumes are discarded to allow for magnetization equilibration. To correct for motion, up to 9 (out of 72) volumes where there was appreciable change in head position between the anatomical acquisition and the BOLD MRI acquisition are excluded before generating maps of CVR. A linear slope of best fit approximates percentage of BOLD signal change per millimeter of mercury change in end-tidal CO_2_. Confidence of this fit is assessed with an *r*-value (Pearson product-moment correlation coefficient).

Cerebrovascular reactivity maps are generated by least squares fitting of the BOLD MRI signal waveform to the P_ET_CO_2_ waveform on a voxel-by-voxel basis. From the fitted data, percentage MRI signal change per millimeter of mercury P_ET_CO_2_ change on a voxel-by-voxel basis is calculated (=CVR). The anatomical images are segmented into GM and WM using statistical parametric mapping software (SPM5, Wellcome Department of Imaging Neuroscience, Institute of Neurology, University College, London, UK), and these masks are used to generate CVR maps.

### Symptom Assessment

Symptoms were assessed using the SCAT2 (20) checklist, a standardized method used to evaluate injured athletes with concussion. This four-page questionnaire comprehensively and quickly assesses current symptoms, with tasks probing cognitive and balance abilities. It enables the calculation of the Symptom Severity Score (SSS) and the Standardized Assessment of Concussion (SAC) score. It should be highlighted that the SCAT2 was designed for “sideline sports concussion” assessments and longitudinal data comparisons with a pre-injury baseline. When used to diagnose (sports) concussion, sensitivity (0.8–0.9) and specificity (0.9–1.0) of the SCAT2 is good, especially for the SSS and SAC components (25). GCS, mechanism of injury, loss of consciousness and duration, initial neurological examination, and imaging results were also recorded. SCAT assessments were performed within 1 day of each MRI.

### Statistical Analysis

Statistical Analysis was performed using SPSS 22. Continuous data are presented as mean ± SD. Paired *t*-tests were used to compare the variables within TBI patients at two time points. Normality of distribution of the data was tested with the Shapiro–Wilk test, and non-parametric tests (Mann–Whitney) were used to compare CVR indexes and volumetric data among the groups. Analysis of covariance including age, sex, and presence of TBI was used to evaluate the influence of each variable on SCAT2 scores. Pearson product-moment correlation coefficient was used to measure the correlation between CVR indexes and SCAT2 scores, SSS scores, age, and sex.

## Results

Patient demographics and mechanism of trauma are shown in Table 1. Mean age was similar between the groups (*p* = 0.39). No difference in sex distribution between groups was observed.

**Table 1:** **Patient demographics and mechanism of trauma**.

		Controls	mTBI
*N*		18	25
Male # (%)		11 (61)	18 (72)
Age μ (±SD)*		38.7 (±12.6)	42.7 (±16.3)
mTBI mechanism	Fall	N/A	10 (40%)
	Assault	N/A	2 (8%)
	MVC	N/A	9 (36%)
	Sports	N/A	3 (12%)
	Blunt (industry)	N/A	1 (4%)
Days to MRI post-TBI (±SD)	First visit	N/A	63.5 days (±42)
	Second visit	N/A	180 days (±38)

Descriptive statistics were calculated for mild TBI and healthy control cohorts. No significant difference in age was found between TBI and healthy control participants. *p = 0.39, two sample *t*-test. Sex distribution was evaluated using a binomial probability test and did not differ between groups.

*MVC, motor vehicle crash*.

Table 2 shows the results of clinical (SCAT2, SSS) and radiological evaluations (MRI CVR indexes, GM, and WM volumes) in controls and mTBI patients at the first clinic visit. Brain volumes were similar between controls and mTBI at visit 1 for both WM (*p* = 0.71) and GM (*p* = 0.36). SSSs and SCAT2 scores were significantly different in controls and TBI in visit 1 (*p* = 0.0001 for both). Although age was negatively correlated to SCAT2 scores for mTBI patients in the first (*r* = −0.62, *p* = 0.0010) and follow-up visits (*r* = −0.46, *p* = 0.048) as well as healthy controls (*r* = −0.6, *p* = 0.007), analysis of variance, including age and sex, showed that mTBI continued to have a significant interaction with SCAT2 scores (mTBI: *p* = 0.00001; age: *p* = 0.02; sex: *p* = 0.13). This difference was still present if age and sex were included in analysis of variance (mTBI: *p* = 0.0003; age: *p* = 0.43; sex: *p* = 0.25).

**Table 2:** **Clinical scores and MRI data in healthy control and mTBI participants at the first clinic visit**.

	Controls	mTBI	*p*
*N*	18	25	
SSS	5.28 (±10.2)	35.72 (±24.8)	0.0001
SCAT2	91.5 (±5.7)	72.76 (±10.9)	0.0001
WM CVR	0.160 (±0.04)	0.1472 (±0.05)	0.28
GM CVR	0.28 (±0.06)	0.26 (±0.091)	0.37
Brain CVR	0.215 (±0.04)	0.20 (±0.072)	0.34
WM volume	417.1 (±47.2)	413.83 (±48.8)	0.71
GM volume	375.2 (±40.8)	365.14 (±47.4)	0.36
CO_2_ levels			
Minimum	34.94 (±3.36)	34.63 (±5.03)	0.82
Maximum	44.0 (±4.04)	44.70 (±3.67)	0.56

*This table describes group differences between symptom and imaging metrics for each group. *p*-values reported are based on Mann–Whitney non-parametric tests*.

Table 3 shows results of the 19 TBI patients who returned for a follow-up visit.

**Table 3:** **Symptom and MRI evaluations of longitudinally followed mTBI patients (*N* = 19)**.

	mTBI visit 1	mTBI visit 2	*p*
Mean days post-TBI	63.5 days (±42)	180 days (±38)	
SCAT2	72.76 (±10.9)	77.8 (±14.22)	0.07
WM CVR	0.1472 (±0.05)	0.1468 (0.40)	0.50
GM CVR	0.26 (±0.091)	0.25 (±0.074)	0.49
Brain CVR	0.20 (±0.072)	0.20 (±0.05)	0.45
WM volume	413.83 (±48.8)	410.97 (±53.9)	0.89
GM volume	365.14 (±47.4)	351.4 (±42.3)	0.005

*Paired *t*-tests were performed to evaluate difference in symptom scores and imaging metrics for TBI patients returning for visit 2*.

**Figure 1:**
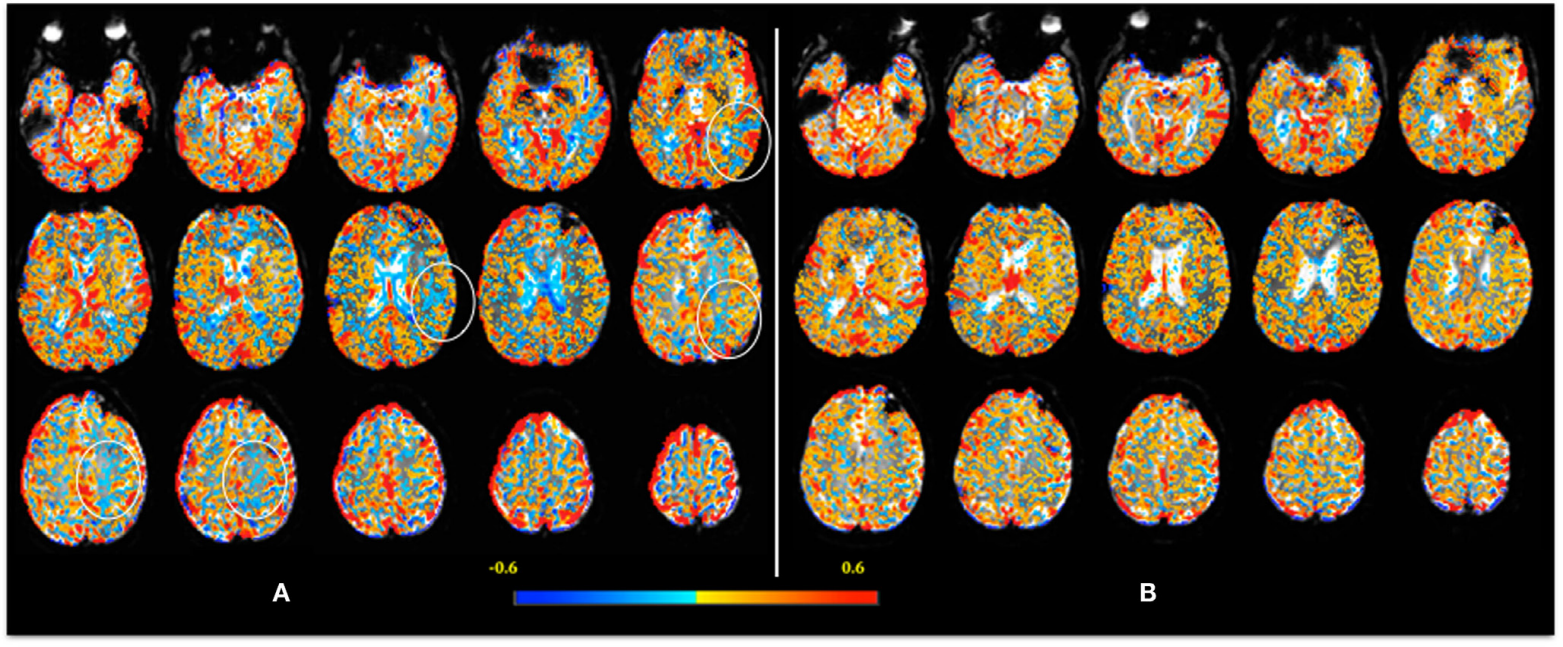
**Example of an individual CVR map 1 week after injury (A)**. CVR is calculated voxel-by-voxel and color-coded, with lower CVR values coded as blue. Note, in **(A)**, the “blue areas” are more evident in the left frontoparietal [where a few regions of interest are highlighted (circles)] and periventricular regions. The decreased CVR in the ventricles is an expected response – please see text for detail. In the follow-up scan **(B)**, 2 months after injury, note the decrease in “blue areas” indicating return of CVR to normal.

In the mTBI cohort, between the first visit and the follow-up, there was a reduction in GM volume (*p* = 0.005) but not WM volume (*p* = 0.89). No difference was detected between early and late mean CVR indexes in the mTBI patients with a follow-up visit (GM: *p* = 0.49; WM: *p* = 0.50; brain CVR: *p* = 0.45), despite noticeable improvement in the CVR maps in some subjects (Figure 1). Table 4 shows where significant correlations were found between symptoms and imaging metrics for the mTBI patients (*n* = 19) with follow-up visits. Only significant correlations are displayed.

**Table 4:** **CVR correlates among TBI patients (*N* = 19)**.

	WM CVR	GM CVR	Whole brain CVR
SCAT2	NS	*r* = 0.49	*r* = 0.48
		*p* = 0.03	*p* = 0.04
WM volume	*r* = −0.46	NS	NS
	*p* = 0.05		
GM volume	NS	NS	NS

*Pearson correlation coefficients were calculated for the mTBI patients (*n* = 19) who completed two visits. Significant correlations are displayed*.

*NS, not significant*.

### SCAT2 Scores and CVR

**Figure 2:**
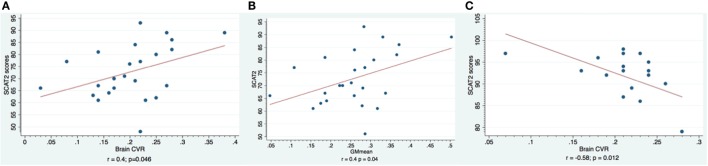
**Correlation between SCAT2 scores and CVR indexes**. Subjects with mTBI are shown in the first two charts. In **(A)**, the whole brain CVR is shown, and in **(B)**, the gray matter CVR is shown. Healthy controls are shown in chart **(C)**: note the negative correlation between CVR values and SCAT2 performance.

A negative correlation between SCAT2 scores and CVR was seen in controls for WM (*r* = −0.59; *p* = 0.01, Pearson), GM (*r* = −0.56; *p* = 0.016, Pearson), and WB (*r* = −0.58; *p* = 0.01, Pearson). Interestingly, in mTBI patients, the correlation between CVR and SCAT2 scores was positive and only present for WB (*r* = 0.4; *p* = 0.046, Pearson) and GM (*r* = 0.4; *p* = 0.05, Pearson) in visit 1. SCAT2 scores were strongly correlated with GM volume (*r* = 0.52, *p* = 0.008, Pearson) in mTBI patients (first visit) but not in controls (*r* = 0.29, *p* = 0.24, Pearson) (Figure 2).

We analyzed the data for the patients with follow-up visit only to confirm that the six patients lost for follow-up did not influence these results. For the 19 patients with CVR MRI at two time points, significant correlations between SCAT2 scores and CVR and brain volumes were demonstrated. Please refer to Tables 3 and 4.

## Discussion

The main findings in this study were that (1) patients with mTBI, even if minimally symptomatic, showed lower SCAT2 scores compared to controls, a finding that persisted in the 180 days after injury; (2) in patients with mTBI, a positive correlation between SCAT2 scores, GM, and brain CVR indexes was seen, while in healthy controls this correlation was negative; this correlation is more evident in the initial test (average 60 days post-injury); and (3) a detectable loss in GM volume in patients was evident in the mTBI patients when comparing early and delayed MRIs.

In our cohort, as expected, patients with mTBI were much more likely to complain of symptoms and performed significantly worse on the SCAT2 test compared to controls during the initial assessment for the study, on average 60 days after injury. Interestingly, although there was a trend for improvement in SCAT2 scores (*p* = 0.06) during follow-up, on average 180 days post-injury, the SSS did not change. Despite performing better, patients were as likely to report and complain of symptoms related to the injury at 6 months than they were at 2 months. Even though most of the reported symptoms are non-specific (e.g., headache, dizziness, and feeling generally “slowed down” cognitively), these patients also scored lower in the examiner driven portion of the test, where the SSS scores are excluded.

Although improved SCAT2 scores of mTBI patients in follow-up were still significantly lower than the controls’ scores, it suggested that the improvement might take longer than expected. This inability to perform well so many months after injury might influence the patient’s perception of their clinical condition, as reflected in similar SSS between the first and follow-up visits.

The primary brain injury related to TBI occurs at the moment of impact, with diffuse axonal injury being the most important primary lesion. Sudden acceleration–deceleration creates shearing forces in the brain that can lead to axonal damage, from minor intra-axonal changes to major dysfunctions in ion transport and intra-axonal flow leading to axon swelling and lysis, proportional to the severity of the injury. With axonal injury, release of excitatory neurotransmitters, microvascular dysfunction and hemorrhage, and edema might ensue (26).

Diffuse axonal injury is a consistent finding in mild, moderate, and severe traumatic head injury, with increasing severity in worse injuries. On the other hand, secondary brain injury develops within hours after impact and mainly consists of ischemia (2), being well recognized as a cause of poor outcome in moderate and severe TBI (27, 28). More recently, it has been suggested that patients with mild injuries might also be at risk of secondary injury (12, 29, 30).

Maintaining adequate CBF is one of the cornerstones of TBI management (31–33), and the importance of cerebral hemodynamics and its disruption leading to hypoperfusion or hyperperfusion situations are well recognized as important mechanisms of further neuronal damage after TBI or stroke. Historically, this has been discussed in the setting of severe TBI, but with the demonstration of altered CBF in milder injuries, it might also be of importance in milder cases.

Diffuse morphological changes, including microscopic axonal injury, have been shown in humans after mTBI (2, 34–36). In a primate model of acceleration injury, Maxwell et al. demonstrated morphological changes in the foot processes of perivascular astrocytes with persistent increase in endothelial projections in the arterioles and venules throughout the brain (37). These morphological changes cannot be visualized even with modern imaging, but it is reasonable to assume that they may lead to alterations in the cerebral microvasculature that could affect cerebral hemodynamics.

Reduction of CBF early after TBI has been well demonstrated after severe TBI (38), and disruption of autoregulation (39, 40) and reactivity to CO_2_ (41, 42) have been shown to correlate with outcome after severe injury. Disturbances in autoregulation (12, 43) and CVR (3, 11) have also been shown in mTBI. Although a link between CVR and clinical symptoms after mild injury has been suggested (11), little is known about the temporal evolution of the CVR impairment, if present, and its anatomical distribution and regional specificity, if any.

Another possible cause for changes in CBF after mTBI is inflammation. Changes in inflammatory cell marker expression and cellular infiltration (44) and increased cytokines (e.g., IL 2, IL 6, and TNF alpha) have been reported after mTBI. Inflammatory cell migration to the venous vasculature and increased leukocyte–endothelium interactions in venules after TBI can result in local inflammatory response, microthrombi formation, and obstruction of venous outflow and also influence the endothelial cells and local production of vasoactive substances, besides potentially increasing vessel wall rigidity, changing venous hemodynamics, and decreasing intracranial compliance (45).

Our results show a correlation between lower SCAT2 scores, indicating poorer performance, and lower CVR indexes, indicative of a blunted or absent response from the microvasculature to CO_2_ changes. Although impairment of CVR after mTBI was demonstrated before (11, 46) the use of MRI imaging allows better anatomical discrimination. We showed that GM CVR is better correlated with symptoms; this finding is in agreement with previous studies using TCD, since TCD is usually used to measure middle cerebral artery (MCA) blood flow velocity, the main supplier of hemispheric GM.

**Figure 3:**
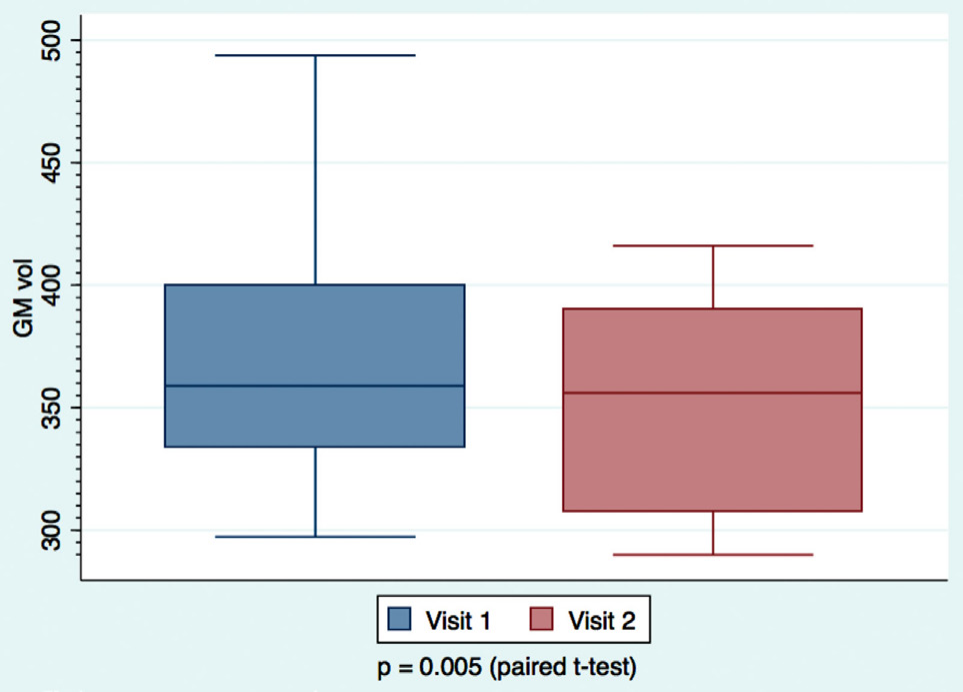
**Mild TBI patients’ gray matter volume measurements calculated at day 63 and day 180**. Paired *t*-tests were performed, with significance set at *p* < 0.05. Larger variability is observed at the earlier visit.

We found a decrease in GM volume between the 2- and 6-month visits, when looking at the mTBI (Figure 3) patients who were longitudinally followed. Van der Naalt et al. found that approximately a third of patients with mild and moderate head injury have focal atrophy in the frontal and temporal regions on MRI in the chronic phase, and that the degree of atrophy is predictive of outcome (47). Metting et al. demonstrated frontal hypoperfusion in the acute phase of mTBI (48). They also report decreased cerebral blood volume (CBV) that correlated with decreased fractional anisotropy (FA) in DTI MRI performed on average 160 days later.

Hofman et al. compared early MR images and SPECT scans after mTBI and found an association between abnormal imaging (MRI and/or SPECT) in the acute phase (less than 5 days) and brain atrophy after 6 months, yet with little correlation to general neurocognitive outcome (49). Interestingly, there was no correlation between MRI and SPECT regarding the presence and location of posttraumatic lesions, even though atrophy was correlated to the presence of lesions in either test. Because the majority of abnormal SPECT findings were related to hypoperfusion, the authors speculate that this could indicate early hypoperfusion that would lead to delayed atrophy.

**Figure 4:**
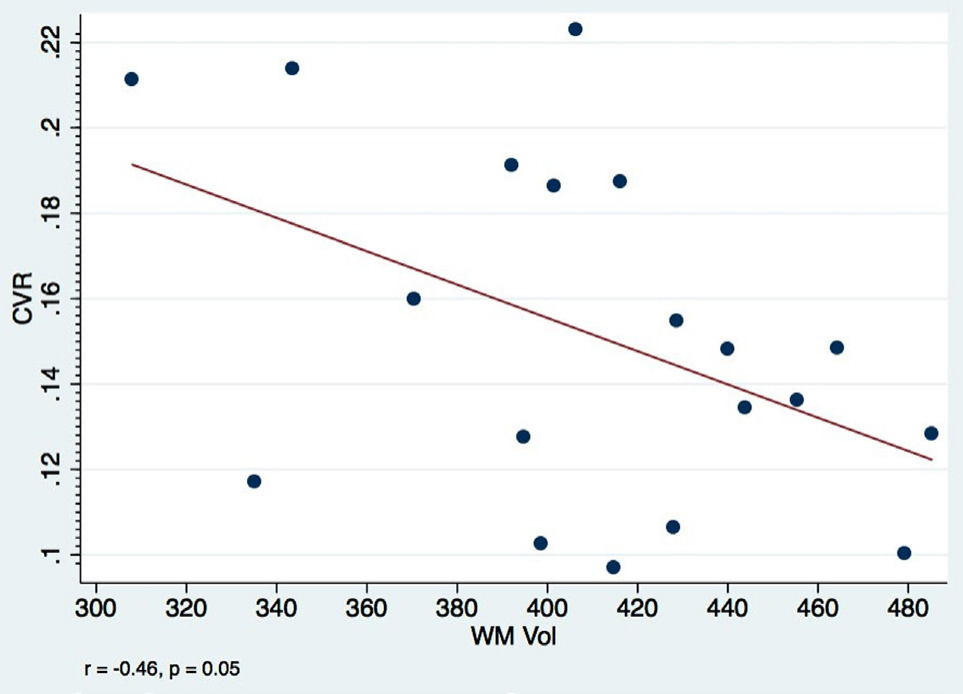
**Visit 1 white matter CVR and volumes: a negative correlation between WM CVR and WM volumes among mTBI patients is observed at only 2 months after injury**. Pearson’s correlation coefficient *r* = −0.46, *p* = 0.05.

In our cohort, none of the patients had parenchymal posttraumatic lesions, and therefore lesion resolution cannot be the underlying process to explain the atrophy. It is possible that all these findings reflect many aspects of the same pathophysiological process where the end result is the decreased perfusion: diffuse microscopic changes in astrocytes and axons in the acute phase of injury lead to dysfunction of the microcirculation and impaired reactivity. One hypothesis is that the microcirculation might be in a state of vasoconstriction due to local changes in astrocyte foot processes and inflammatory reaction, which might result in decreased in CBV and eventual atrophy. The negative correlation (Figure 4) found between WMCVR and WM volume might offer further support to the theory of vasoparalysis: more affected vessels, without capacity of vasodilatation, will not increase in diameter with increasing levels of CO_2_, resulting in lower WM volumes. The lack of CVR reactivity would be a marker for more severe microstructural injuries within the mTBI spectrum.

One of the advantages of using prospective targeting to CO_2_ levels is that stimulation of the vasculature is independent of neurovascular coupling. Disruption of coupling is well described after TBI (50, 51), and since BOLD MRI is based ultimately on the metabolic changes, often following increased neuronal activity that results in alteration of CBF and the proportions of oxy and deoxyhemoglobin, loss of neurovascular coupling has the potential to impact BOLD results. By using a method to manipulate CO_2_ directly, we can measure BOLD responses that are independent of neuronal activity. Accordingly, none of our subjects were asked to perform any specific task, which theoretically should decrease the influence coupling could have on our findings. Furthermore, recently Maggio et al. showed that hypercapnia affects neurovascular coupling, but it does not change CVR (17).

Glushakova et al. characterized the acute phase of microvascular damage in TBI when breakdown of the blood–brain barrier, leukocyte translocation, and microglia activation can be seen, followed later by chronic changes, including micro-hemorrhages, iron deposit, and inflammation, and suggest that this process is initiated by mechanical injury to the microcirculation (52). It is possible that one of the phenotypes of this injury is the loss of the capacity to react to changes in CO_2_. The functional disruption of neuronal networks can lead to delays and disorganization of neuronal activity, as demonstrated in electrophysiological studies using magnetoencephalography in mTBI patients (4, 53).

We were surprised by the opposite correlation between SCAT2 scores and CVR in mTBI and healthy controls. In mTBI patients, CVR indexes were on average lower (0.20 ± 0.072) compared to controls (0.22 ± 0.04), but the difference was not significant. TBI patients with lower CVR indexes had the lowest SCAT2 scores and healthy individuals with the highest CVR indexes had the lowest SCAT2 scores in each group, respectively. It is well established that both hypoperfusion and hyperperfusion can cause harm, and we speculate that an “ideal range” for CVR might exist and individuals below or above that range may not be able to perform at their best. This observation has to be considered keeping in mind that the test used to evaluate performance is limited, and in most cases used for sideline assessment of acute concussion only.

Our paper has limitations. We acknowledge the small numbers and recognize that results may not be generalizable to the mTBI population, especially the elderly. Six patients were lost to MRI follow-up. This might have resulted in selection bias where patients with more symptoms were more likely to return. However, we compared the results between the mTBI patients with and without follow-up visit, and the groups were similar (Table 5). We did not include CSF in the analysis, and Figure 1 demonstrates that reactivity tends to be negative (“blue”) in the ventricles. It has been shown that the BOLD time course from the ventricles display inverse correlation with the partial pressure of end-tidal CO_2_ (PetCO_2_) in normal healthy volunteers, likely caused by dilatation of the ventricular blood vessels, which displaces CSF, generating the apparently negative CVR within the ventricular system (54). Further analyses, beyond the scope of this initial work, would include correlations among patients with confirmed DAI on MRI.

**Table 5:** **Comparison of clinical scores and MRI data within mTBI patients after early visit (60 days)**.

	mTBI no follow-up	mTBI with follow-up	*p*
*N*	6	19	
Male # (%)	5 (80)	13 (69)	0.64
Age μ (±SD)	39.5 (±17.7)	43.7 (±16.35)	0.59
Mean days after trauma μ (±SD)	59.7 (±36.12)	64.9 (±45.57)	0.79
SSS	37 (±14.9)	35.3 (±27.6)	0.88
SCAT2	73.3 (±14.7)	72.6 (±9.94)	0.88
WM CVR	0.14 (±0.08)	0.15 (±0.04)	0.52
GM CVR	0.24 (±0.16)	0.26 (±0.066)	0.66
Brain CVR	0.19 (±0.11)	0.20 (±0.01)	0.58
WM volume	423.3 (±55.5)	410.8 (±47.7)	0.59
GM volume	364.3 (±67.15)	365.4 (±41.7)	0.96

*This table is included to highlight the homogeneity within the sample of TBI patients. The subset of six patients who were not able to complete a follow-up MRI or clinic visit show comparable values on symptom scores and imaging indices. These comparisons examine the data collected at visit 1 only*.

Traumatic brain injury is more prevalent in young males, and accordingly, we had more males in mTBI group. The expected frequency of females in the mTBI group, based on the control group numbers, would be 15. Sex differences in CVR have been reported in healthy individuals, with women demonstrating slightly higher reactivity indexes (55). Due to logistic issues, there is a large variation in the time between injury and first scan. This might have affected our results since changes in reactivity and autoregulation after injury are likely to be a dynamic process.

As mentioned above, we used SCAT2 questionnaire. The questionnaire is not a comprehensive test and does not replace neuropsychological testing. However, written neuropsychological tests are time-consuming and require trained personnel, and while computer-based tests are more user friendly, there is no consensus on effectiveness (56). Also, it is a tool designed to diagnose concussion in the acute/subacute period and is usually collected longitudinally. Its use in a more chronic phase after head injury might not be ideal. However, because the SCAT2 is designed for use as a longitudinal follow-up tool, in this case, we felt it would be an appropriate method to measure evolution of posttraumatic symptoms. Our choice of the SCAT2 [which includes balance error scoring system (BESS) – a sensitive test for concussion diagnosis] was based on it being easy to apply, widely used, and easily reproducible, thus facilitating testing and re-testing.

## Conclusion

Our findings suggest that there is a relationship between the impairment of CVR to CO_2_ and performance on the SCAT2 score in patients with mTBI. We also noticed that symptoms and CVR impairment could be seen even after 6 months from an apparently very minor injury. These findings suggest that the reactivity to CO_2_ might be developed into an imaging marker for mTBIs, especially if longitudinal follow-up is feasible.

We also noticed significant decrease in GM volume in follow-up imaging. This has been demonstrated in more severe injuries or milder injuries with evidence of parenchymal lesions in acute imaging. Our findings suggest that even the mildest injuries, without acute findings in CT and/or MRI, might lead to brain atrophy. Although the test in its current state might not be useful to detect the acute concussion in an ER setting, it might be to monitor sports injuries and head trauma in other more controlled environments, offering a more objective measure of effects of minor injuries in brain physiology.

## Author Contributions

LC conceived the project, wrote the protocols, supervised data collection and analysis, and wrote the manuscript. CN and DC helped with data collection (CVR maps) and analysis and revised the protocol draft. JF helped to write the protocol, helped with data analysis, and revised the protocol draft. AB helped with data collection and storage, analysis, and drafting of the initial manuscript.

## Conflict of Interest Statement

The authors declare that the research was conducted in the absence of any commercial or financial relationships that could be construed as a potential conflict of interest.
